# Telehealth in Australia

**DOI:** 10.1192/j.eurpsy.2022.1457

**Published:** 2022-09-01

**Authors:** V. Lakra

**Affiliations:** Royal Australian and New Zealand College of Psychiatrists, President, Melbourne, Australia

**Keywords:** videoconferencing, Covid-19, TeleHealth, telepsychiatry

## Abstract

**Introduction:**

There is a significant psychiatry workforce shortage in Australia, particularly in rural and remote communities. Given the large distances involved, telehealth – providing consultation via videoconference – has been widely accepted. Psychiatrists were among the highest users of telehealth services in Australia before the COVID-19 pandemic. However, the outbreak of COVID-19 resulted in a major transformation to service delivery across Australia. Private psychiatrists and state public mental health services had to rapidly transition to largely telehealth delivery to ensure continuity of care for consumers.

In March 2020, additional telehealth item numbers were added to the Australian Medicare Benefits Schedule (MBS) to encourage physical distancing for those accessing medical services during the pandemic.

**Objectives:**

To provide an overview of the increase in telehealth activity since the COVID-19 pandemic.

**Methods:**

The MBS is the list of services for which the Australian Government will pay a rebate. Key data on MBS telehealth activity since March 2020 was examined.

**Results:**

The use of telehealth has increased during the pandemic. A survey of Royal Australian and New College of Psychiatrists (RANZCP) psychiatrists found that 93% supported retention of telehealth MBS item number numbers following the COVID-19 pandemic, noting increased accessibility for consumers. Positive feedback has been received from consumers.

**Conclusions:**

During 2020 and 2021, the RANZCP worked with the Australian Government to ensure there were appropriate MBS telehealth services available for consumers. The RANZCP continues to work with the Government as they plan for a longer-term transformation of telehealth services beyond 2021.

**Disclosure:**

No significant relationships.

